# Origin, evolution, breeding, and omics of Apiaceae: a family of vegetables and medicinal plants

**DOI:** 10.1093/hr/uhac076

**Published:** 2022-04-11

**Authors:** Xiao-Jing Wang, Qing Luo, Tong Li, Ping-Hong Meng, Yu-Ting Pu, Jie-Xia Liu, Jian Zhang, Hui Liu, Guo-Fei Tan, Ai-Sheng Xiong

**Affiliations:** 1 Key laboratory of Plant Resource Conservation and Germplasm Innovation in Mountainous Region (Ministry of Education), Guizhou University, Guizhou 550025, China; 2 Institute of Horticulture, Guizhou Academy of Agricultural Sciences, Guizhou 550006, China; 3 State Key Laboratory of Crop Genetics and Germplasm Enhancement, Ministry of Agriculture and Rural Affairs Key Laboratory of Biology and Germplasm Enhancement of Horticultural Crops in East China, College of Horticulture, Nanjing Agricultural University, Nanjing 210095, China; 4 College of Agronomy, Jilin Agricultural University, Changchun 210095, China

## Abstract

Many of the world’s most important vegetables and medicinal crops, including carrot, celery, coriander, fennel, and cumin, belong to the Apiaceae family. In this review, we summarize the complex origins of Apiaceae and the current state of research on the family, including traditional and molecular breeding practices, bioactive compounds, medicinal applications, nanotechnology, and omics research. Numerous molecular markers, regulatory factors, and functional genes have been discovered, studied, and applied to improve vegetable and medicinal crops in Apiaceae. In addition, current trends in Apiaceae application and research are also briefly described, including mining new functional genes and metabolites using omics research, identifying new genetic variants associated with important agronomic traits by population genetics analysis and GWAS, applying genetic transformation, the CRISPR-Cas9 gene editing system, and nanotechnology. This review provides a reference for basic and applied research on Apiaceae vegetable and medicinal plants.

## Introduction

Apiaceae contains 434 genera and nearly 3780 species, including many important vegetables, such as carrot (*Daucus carota*), coriander (*Coriandrum sativum*), and celery (*Apium graveolens*) [1], which are mainly distributed in northern temperate regions [2]. Apiaceae also contains important medicinal plants, including *Angelica sinensis*, *Peucedanum praeruptorum*, and *Angelica dahurica*, which are aromatic herbs with alternating feathered leaves that are sheathed at the base of a shortened stem [3]. The flowers of Apiaceae plants are usually bisexual and include five sepals and petals, as well as an enlarged disk at the base of the style, and form a conspicuous flat-topped umbel [4, 5]. The cremocarp consists of two parts that split open in Apiaceae seeds [6].

Previous studies have revealed that the Apiaceae family is rich in secondary metabolites that have medicinal value [7, 8]. The Apiaceae family includes many vegetable crops that are rich in flavonoids, carotenoids, coumarin, coumarin derivatives, vitamins, and minerals [8], such as celery, carrot, parsley (*Petroselinum crispum*), and fennel (*Foeniculum vulgare*) [9]. Apiaceae plants are also used as herbs and spices, including dill (*Anethum graveolens*), coriander (*Coriandrum sativum*), caraway (*Carum carvi*), and cumin (*Cuminum cyminum*) [10, 11]. Moreover, some species were used as herbal folk remedies in ancient times, including gum ammoniac (*Dorema ammoniacum*), goutweed (*Aegopodium podagraria*), *Peucedanum luxurians*, and *Seseli devenyense* [12–15]. Some Apiaceae species are grown as ornamental flowering plants, such as masterwort (*Astrantia*) [16], blue lace flower (*Trachymene caerulea*) [17], and sea holly (*Eryngium maritimum*) [18]. In addition, the Apiaceae family includes many toxic perennial plants, such as poison hemlock (*Conium maculatum*), which contains the toxin coniine [19, 20]_,_ water hemlock (*Cicuta maculata*), which contains the toxin cicutoxin [21], and fool’s parsley (*Aethusa cynapium*), which contains the toxin coniine [22, 23]. The major Apiaceae vegetable species and medicinal species are summarized in Table 1.

**Table 1:** Medicinal applications of major compounds in Apiaceae vegetable and medicinal species.

**Common name**	**Latin name**	**Edible parts**	**Main compounds**	**Use**
Anise	*Pimpinella anisum*	Seed	*Trans*-anethole, *p*-anisaldehyde, estragole, farnesol, limonene, 4′-methoxypropiophenone [24, 25]	Edible
Asafoetida	*Ferula assafoetida*	Root	Quercetin, gallic acid, phenol, arsine triethyl, 8-acetoxy-5-*S*-hydroxyumbelliprenin, asadisulfide, vanillin, β-sitosterol [26, 27]	Antifungal, antidiabetic, anti-inflammatory, antimutagenic, antiviral [28]
Bei Chaihu	*Bupleurum chinense*	Root	Saikosaponin-D, 1-*O*-caffeoylglycerol, esculetin, scopoletin, α-spinasterol [29, 30]	Antioxidant, hepatoprotective, anti-inflammatory, antipyretic, analgesic, immunomodulatory [31, 32]
Caraway Carrot	*Carum carvi Daucus carota*	Seed, root	Limonene, carvacrol, carvone, carvenone, linalool, *p*-hydroxybenzoic acid, kaempferol, naringenin [33, 34], β-carotene, quercetin, luteolin, kaempferol, myricetin [35, 36]	Antispasmodic, carminative, astringent [33], edible
Celery	*Apium graveliens*	Petiole, leaves	Apigenin, luteolin, kaempferol, caffeic and ferulic acids [37, 38]	Edible
Chinese angelica	*Angelica sinensis*	Root	Ferulic acid, *Z*-ligustilide, *Z*-butylidenephthalide, *N*-butylidenephthalide, *E*-ligustilide, *p*-hydroxybenzoic acid [39–41]	Anti-inflammatory, immunostimulatory, anticancer, neuroprotective, antihepatotoxic, antioxidative，anticardiovascular [42]
Chuanminshen	*Chuanminshen violaceum*	Root	Bergapten, ficusin, 2,3-dihydro-3,5-dihydroxy-6-methyl-4H-pyran-4-one, falcarinol [43]	Antioxidant, immunomodulatory, anti-inflammatory, antitussive [44, 45]
Cnidium	*Cnidium monnieri*	Fruit	Osthol, osthenol, imperatorin, isopimpinellin, bergapten, xanthotoxol, isoporalen, isopimpinelline [46, 47]	Anthelmintic, anti-allergic, anti-atherosclerosis, analgesic, antibacterial [48]
Coral vegetable	*Glehnia littoralis*	Root	α-Pinene, limonene, β-phellandrene, germacrene B, spathulenol [49]	Antioxidant, antitumor, anti-amnesic, immunomodulatory, antimicrobial, allelopathic [50, 51]
Coriander	*Coriandrum sativum*	Stem, leaves	β-Carotene, β-cryptoxanthin epoxide, lutein-5,6-epoxide, violaxanthin, neoxanthin [52]	Edible
Cumin	*Cuminum cyminum*	Seed	*p*-Cymene, thymoquinone, α-thujene, gallic and vanillic acids, luteolin, catechin, coumarin, eugenol [53, 54]	Edible
Dill	*Anethum graveolens*	Seed	Carvone, *trans*-dihydrocarvone, dill ether, α-phellandrene, limonene [55]	Edible
Dwarf pennywort	*Hydrocotyle sibthorpioides*	All	Quercetin, isorhamnetin, 6-caffeoylgalactoside, stigmasterol, daucosterol [56]	Antihyperglycemic, antioxidant, antitumor [57, 58]
Fennel	*Foeniculum vulgare*	Stem, leaves, seed	*Trans*-anethole, estragole, fenchone, limonene, rosmarinic acid [59]	Edible
Ferula	*Ferula sinkiangensis*	Stem, leaves	Coumarins, sesquiterpenes, sesquiterpene lactones, sesquiterpene coumarins, glucuronic acid [60]	Antineuroinflammatory, antibacterial, antimicrobial, anti-inflammatory, anticancer, antioxidant, antileishmanial [61]
Gotu kola	*Centella asiatica*	All	Chlorogenic acid, madecassoside, asiaticoside, madecassic acid, asiatic acid [62]	Antidiabetic, wound-healing, antimicrobial, memory-enhancing, antioxidant, neuroprotecting [63]
Japanese parsley	*Cryptotaenia japonica*	Stem, leaves	Luteolin, apigenin, *p*-coumaric acid, caffeic, ferulic acid [64]	Antioxidant, antibacterial, anti-inflammatory [64]
Lovage	*Levisticum officinale*	Leaves	Falcarinol, (*Z*)-ligustilide, (*Z*)-3-butylidenephthalide, *trans*-β-farnesene, β-phellandrene [65]	Edible
Notopterygium	*Notopterygium incisum*	Stem, root	Notopterol, bergapten, imperatorin, isoimperatorin, cnidilin, pabulenol, alaschanioside C [66, 67]	Analgesic, antioxidant, anti-inflammatory, antiviral, anti-arrhythmic, immunosuppressive [66]
Parsley	*Petroselinum crispum*	Petiole, root, stem, leaves	Apigenin, phenylpropanoids apiol, oleanolic acid, furanocoumarins, isoimperatorin, oxypeucedanin [68, 69]	Antioxidant, hepatoprotective, brain protective, antidiabetic, analgesic, spasmolytic, immunosuppressant, antiplatelet [70]
Parsnip	*Pastinaca sativa*	Leaves, root	Xanthotoxin, bergapten, isopimpinellin, imperatorin [71]	Edible
Radix changii	*Changium smyrnioides*	Root	4-Methoxycinnamic acid, 7-hydroxy coumarin, cafeic acid, α-terpinene, β-patchoulene [72]	Antitussive, eliminating phlegm, anti-asthmatic, immunoregulatory, antioxidant, antitumor, antifatigue, antihypoxia, anti-atherosclerotic [72]
Slender celery	*Apium leptophyllum*	Seed	β-Sitosterol, apigenin, quercetin, luteolin, kaempferol, isorhamnetin, β-selinene, *p*-cresyl *iso*-valerate [73–75]	Edible
Water Dropwort	*Oenanthe javanica*	Petiole, leaves	Persicarin, apigenin, isorhamnetin, quercetin, hyperoside, azelaic acid, myristic acid, catechol, 3,5-dihydroxybenzoic acid [76, 77]	Edible
Water dropwort	*Ostericum sieboldii*	Petiole, leaves	Myristicin, α-terpineol, α-cadinol, β-farnesene, linalool [78]	Analgesic, anti-inflammatory [78]
Wild chervil	*Anthriscus sylvestris*	Stem, leaves, root	β-Phellandrene, *Z*-β-ocimene, α-pinene, (−)-deoxypodophyllotoxin, chlorogenic acid, luteolin-7- *O*-glucoside, isoflavone, picropodophyllotoxin, falcarindiol [79–83]	Antitumor, antimicrobial, anti-inflammatory, antioxidant [80]

In recent years, Apiaceae plants have been studied with respect to bioactive compounds, medicinal applications, omics, and traditional or modern separation techniques for rare compounds. Many important substances and mechanisms of Apiaceae plants have been fully revealed, and summarizing these research advances can further promote the application of Apiaceae plants. In this review, we summarize the complex origins of Apiaceae and the current state of research on the family, including traditional and molecular breeding practices, bioactive compounds, medicinal applications, omics research, molecular markers, regulatory factors, functional genes, genomics research, functional gene mining, and molecular breeding, and discuss future perspectives.

## Apiaceae origin

### The complex origins of vegetables and medicinal plants in Apiaceae

Although >70% of Apiaceae family genera are distributed in the Northern Hemisphere [84], biogeographical and molecular phylogenetic studies demonstrated that the Apiaceae family originated in the Southern Hemisphere [84, 85]. Furthermore, Australasia was estimated to be the place of origin of crown Apiaceae plants during the early Paleogene [86].

The Apiaceae family has been mainly divided into four subfamilies: Azorella, Centella, Apioideae, and Eryngium [87, 88]. Apioideae subfamilies include several important vegetable crops: celery, carrot, parsley, water dropwort, and coriander [89–91]. However, each of these Apiaceae species has distinct origins. Carrot and celery originated in Middle Asia around Afghanistan [8, 75, 92], and slowly spread into the Mediterranean area [93]. The earliest recorded carrots were mainly purple or yellow, with some white or black species, instead of orange [94]. Parsley originated in the late third century BC on the Mediterranean coast [95], where it was used for decoration and seasoning [96]. Water dropwort originated in Europe and the Mediterranean region, whereas coriander originated in the Middle East region [97, 98]. The Apiaceae family also contains many important Chinese herbal plants [99]; the origins of many of these plants remain unclear. For example, recent studies revealed that the *Angelica* group has been cultivated for food and medicine since at least 800 AD [100], and originated in the Middle East [101], possibly Syria, or northern European countries [102]. Although the Apiaceae family contains many species, most of the members of this family have not been comprehensively investigated, especially vegetables and medicinal species.

### Bioactive compounds in vegetables and medicinal plants in Apiaceae

All vegetables and medicinal species in Apiaceae have effective secretory systems involving different organs, including roots, stems, leaves, flowers, and fruit [103–105]. According to previous studies, the biologically active compounds of Apiaceae plants can be divided into two groups: nutrients and nutraceuticals [106]. Nutrients are important plant growth regulators that mainly include minerals, proteins, fiber, carbohydrates, and lipids [107]. In contrast, nutraceuticals, a portmanteau word derived from ‘nutrition’ and ‘pharmaceutics’, are non-nutritive plant compounds with high antioxidant activity [108–110]_._ Nutraceuticals_,_ which mainly include polyphenolic compounds, polyacetylenes, and terpenoids [106], are thought to promote health and are used in the food processing and pharmaceutical industries [111–113].

### Phenolic compounds

Phenolic compounds, such as phenolic acids, simple phenols, flavonoids, and hydroxycinnamic acid derivatives [114], are responsible for the flavor, color, and sensory properties of plant-derived foods and beverages [112, 115], and they also contribute to the nutritional qualities of vegetables and medicinal plants [116]. Several studies have pointed out the value of phenolic compounds in some Apiaceae plants [117–119]. The phenolic compounds, such as flavonoids, phenolic acids, coumarin and tannins in fennel, apiin and malonylapiin in parsley, and apiin in celery, are responsible for organoleptic characteristics, such as bitterness, astringency, color, flavor, and odor [117]. The antioxidant activity of many Apiaceae plants has also been attributed primarily to phenolic compounds [120, 121]. Celery contains the flavonoids apigenin, luteolin, kaempferol, isorhamnetin, and quercetin, and extracts of celery have antibacterial, anti-inflammatory, antioxidation, antitumor, and cardiovascular protective activities [75]. The luteolin-7-*O*-β-d-glucoside (cynaroside) from *Anthriscus sylvestris* displays biological activity, especially against Gram-negative bacteria, exhibits antimutagenic activity, suppresses biofilm formation of *Pseudomonas aeruginosa* and *Staphylococcus aureus*, and increases the frequency of mutations leading to ciprofloxacin resistance in *Salmonella typhimurium* [122]. Moreover, phenolic compounds can be used to extend the shelf life of foods, delay the oxidation of inclusions [123], and reduce the risk of cancer and cardiovascular, cerebrovascular, and nervous system diseases [124]. Ferulic acid from *Angelica sinensis* and *Ferula teterrima* exhibited a therapeutic effect on membranous nephropathy-induced proteinuria and breast cancer [125, 126]. In addition, the content of various bioactive substances in plants is regulated by many factors, including environment, cultivation techniques, varieties, and harvest time [127–129].

### Polyacetylenes

Recent studies demonstrated that several polyacetylenes isolated from Apiaceae plants have high toxicity to bacteria, fungi, and mammalian cells [130–132], as well as neurotoxicity, an inhibitory effect on platelet aggregation, and the potential to cause allergic skin reactions [131]. In mammalian experiments, polyacetylenes inhibited tumor formation [131, 133], indicating that these compounds may have clinical applications. A group of aliphatic C17 polyacetylenes in carrot, celery, parsley, and parsnip have revealed interesting antitumor (namely antileukemic), anti-inflammatory and antiplatelet aggregatory effects in mammals [134]. However, polyacetylenes have a negative impact on the taste of the roots of Apiaceae vegetables and medicinal plants, such as parsnip, celeriac, parsley, carrot, and fennel bulbs, because they increase bitterness [135]. In addition, some conjugated polyacetylenes (cicutoxin, oenanthotoxin, virol A, virol B, and virol C) produced by species of the *Oenanthe* (*O. crocata*) and *Cicuta* genera (*C. virosa*, *C. maculata*, and *C. douglasii*), have been identified amongst the strongest plant neurotoxins [134].

### Terpenoids

Terpenoids, such as aromatic and essential oils, are the largest group of specialized metabolites in plants [136]. Some terpenoids are specifically distributed in the Apiaceae plants, such as carotol in carrot, *trans*-anethole in anise and fennel, and carvone in caraway and dill. These species are commonly used as food supplements for their aromatic qualities, which can enhance the smell and taste of foods [137, 138]. Terpenoids possess antioxidant and antimicrobial activities and are the main components of essential oils [139]. The antioxidant effects of terpenoids have led them to be used to improve and treat some diseases, such as cancer, cirrhosis, rheumatoid arthritis, and arteriosclerosis [140]. Asiaticoside in *Centella asiatica* suppressed the viability of colorectal cancer and increased cell apoptosis by inhibiting the activation of the NF-κB signaling pathway by downregulating IκBα phosphorylation [141]. Moreover, the antimicrobial activities of terpenoids have led them to be used to make efficient antibiotics and antimycotic agents [142, 143].

### Applications of edible organs from representative Apiaceae species

The purpose of this review is to investigate edible organs containing nutraceuticals and having medicinal value in representative plants of the Apiaceae family. Nutraceuticals and medicinal value in the Apiaceae family play an important role in food safety and have health benefits [144, 145]. The seed is the characteristic reproductive body of both angiosperms and gymnosperms. The seeds of Apiaceae plants, which are actually dried fruit, are used as natural food additives for spices and seasonings [146]. Acimovic and Milic [103] have summarized the types and uses of nutraceuticals in 12 Apiaceae plants in detail. For example, the dried fruit of *Apium graveolens*, *Carum sativum*, and *Foeniculum vulgare* are used in salads, cakes, sausages, curries, soups, vegetables, and other foods [103]. Moreover, essential oil may be extracted from the seeds of some plants of the Apiaceae family [118]. For example, *Carum carvi*, *Petroselinum crispum*, *Cuminum cyminum*, and *Daucus carota* may be used to produce essential oils for use in food processing [8], and as additives in candy, chewing gum, soft drinks, and beer [147]. Parsnip has a sweet taste similar to nutmeg and cinnamon, as well as a unique aromatic character [148]. Previous studies have revealed that the seeds of other vegetables and medicinal plants in Apiaceae, such as dill, coriander, and fennel, also contain many different types of nutraceuticals with important medicinal value at varying concentrations [149]. For example, dill seeds are used to relieve colic pain and treat diarrhea, asthma, neuralgia, diabetes, cardiovascular diseases, gallbladder disease, and other conditions [150, 151]. Cumin seeds have been used widely in traditional Chinese medicinal practices to treat toothaches, diarrhea, epilepsy, dyspepsia, and jaundice [152]. Carrot seeds were shown to improve memory when administered to Alzheimer’s patients and were found to have hypoglycemic and hypolipidemic properties [153].

The leaves, stems, and roots of Apiaceae family plants are important vegetative organs that are used for pickling, as well as being consumed fresh. The fresh leaves of dill, coriander, parsley, and celery are used in many countries as garnishes and to flavor salads, dips, snacks, and soups [103, 154]. Petioles, such as celery, are used for the preparation of salads, juices, soups, stews, and sauces [155]. The roots of Apiaceae members are used as food and medicine. For example, *Angelica* and lovage roots are used to flavor meat and canned vegetables, but they can also be used as raw material for the production of herbal liqueurs and bitter spirits [106, 156]. The fresh roots of carrot and parsnip are the most widely consumed Apiaceae root vegetables, and they are primarily eaten raw in juices or salads, or for pickling, soups, and cakes. More importantly, carrot taproots contain effective anti-inflammatory and anticancer compounds, as well as constituents with hypoglycemic and hypolipidemic properties [157].

## Genetic breeding

### Male sterile breeding

Genetic male sterility and cytoplasmic–nuclear male sterility (CMS) have been utilized for the production of hybrid cultivars in Apiaceae [158]. A male-sterile line was also used in the mechanization of hybrid seed production, which simplified the procedure and reduced its cost [159, 160]. The difficulty of obtaining *F*_1_ hybrid seeds in vegetables and medicinal plants in Apiaceae is due to a lack of emasculation methods. Thus, male-sterility breeding is used for Apiaceae crop breeding, including carrot, celery, coriander, and others [161]. Male sterility can be genetically or/and cytoplasmically determined [162, 163]. Currently used cytoplasmic male sterility (CMS) systems include the ‘brown anther’ type and ‘petaloid’ type [164]. The progress of carrot male sterile breeding research were summarized by many researchers in various years [165–167].

*F*_1_ hybrid celery seeds are difficult to obtain, because celery flowers are small, numerous, and easily self-pollinate [163]. The first sterile male celery line was the Iranian accession P1229526, in which sterility is conferred by a single recessive *ms-1* gene [168]. Furthermore, Quiros *et al*. [168] found unstable celery CMS in an unidentified wild celery plant, and Gao *et al*. [169] identified a sterile male celery plant (01-3A) from the inbred line 01-3.

### Disease resistance breeding

Three main methods, (i) selecting disease-resistant varieties, (ii) strengthening cultivation management, and (iii) applying fungicides, are commonly used to prevent and control the occurrence and spread of plant diseases [170]. Selection of disease-resistant germplasm resources has been the most effective method of reducing the occurrence of diseases in vegetables and other food crops [171].

Numerous studies have shown that powdery mildew infects a wide range of Apiaceae plants, including carrot, parsnip, celery, dill, and fennel [172]. Powdery mildew (*Blumeria graminis* f. sp. *hordei*) mainly occurs in leaves and petioles, and can cause fatal damage to Apiaceae vegetable crops. The first report of powdery mildew in Apiaceae vegetables was a report about carrot and parsley crops in the state of Washington in the USA [173]. In addition, *Alternaria radicina*, a seed-borne fungal disease, can decrease seed quality [174].

Early blight, caused by *Cercospora apii*, is a highly transmissible disease of *Apium* [175]. The celery ‘Floribelle M9’ cultivar with superior resistance to early blight was developed in the 1990s and used to develop early blight-resistant cultivars, such as ‘FBL 5-2 M’ [169]. Late blight, caused by *Septoria apiicola*, is an important leaf disease that infects celery, celeriac, and carrot [176]. In addition, two *Septoria*-resistant celery species (*Apium chilense* and *A. panul*) have been crossed to generate plants with enhanced disease resistance [177].

*Fusarium oxysporum* is a soil-borne fungus that causes fusarium yellows disease in celeriac, celery and carrot [169]. UC1 is a fusarium yellow disease-resistant celery breeding line that has been backcrossed with elite varieties to create the resistant lines UC8-1, UC10-1, and UC26-1 [178]. Somaclonal variation has been used to select *Fusarium*-resistant celery plants, such as the MSU-SHK5 line, during regeneration from cell suspensions [179]. In 2017, three potentially resistant celeriac accessions from Turkey and an additional resistant accession from China were identified as sources of *F. oxysporum* resistance [180].

Leaf blight, caused by *Alternaria dauci*, is a fungal leaf disease that negatively impacts carrot and coriander cultivation [174, 181]*.* Gugino and colleagues [182] research identified five carrot cultivars (‘Bolero’, ‘Carson’, ‘Calgary’, ‘Ithaca’, and ‘Fullback’) with relatively low susceptibility to *A. dauci*, as well as three cultivars (‘Bolero’, ‘Carson’, and ‘Bergen’) that showed relatively low susceptibility to *Cercospora carotae.* However, carrot cultivar ‘Fontana’ was found to be highly susceptible to these two diseases [182]. Infection of coriander plants becomes apparent when they bloom, the flowers turn yellow and are generally taller than those of uninfected plants [183]. Moreover, infection of coriander plants becomes apparent when they bloom, the flowers turn yellow, and the plants are generally taller than uninfected plants [184].

Sclerotinia disease, caused by *Sclerotinia sclerotiorum*, *S. minor*, and *S. trifoliorum*, can cause severe damage to stored Apiaceae vegetables, especially carrot [185, 186]. Jensen *et al*. [186] revealed that *Daucus carota*, as a susceptible host to *Sclerotinia sclerotiorum*, can obtain disease-resistance genes from disease-resistant cultivated species during flowering to produce resistant offspring. Although sclerotinia disease also occurs in celery and parsley, the impact on these species is minimal. Aster yellows, caused by a bacterium-like organism called a phytoplasma, is a common destructive disease worldwide [184].

Celery mosaic virus (CeMV) is transmitted by aphids and is the most common viral disease in celery [187]. A single recessive locus and markers linked to CeMV resistance genes were identified in 2001 [188]. Using post-transcriptional gene silencing technology, previous studies attempted to produce celery and carrot plants with resistance to CeMV and carrot virus Y (CarVY), but resistant celery plants were not obtained [169].

Root-knot nematodes (RKNs, *Meloidogyne* spp*.*) are major pathogens that affect carrot [189] and other Apiaceae species, including celery [190] and parsnip [191]. The roots of carrot plants infected by RKNs displayed malformed, stubby, hairy roots with tough galls and thick skin. Moreover, the aerial parts of infected plants become yellow and display inhibited growth and development [192]. At the same time, wounds produced by RKNs on carrots increase the probability of infection by diseases and other pests. RKNs show strong adaptability and can adapt to complex and variable environments. The best carrot RKN-resistant varieties obtained so far include ‘Brasilia’ and ‘Tropical’, and two resistance genes (*Mj-1* and *Mj-2*) have been identified [193–196]. Another resistance locus was identified in the ‘PI652188’ cultivar in 2014 and mapped to a different position in chromosome 8 [189]. Furthermore, RKNs also affect production of fennel; infection increases the size of root galls, decreases plant vigor, and causes a yellow phenotype [197]. Other methods of reducing nematode populations in the soil include solarizing the soil and crop rotation [198].

### Breeding for insect pest resistance

The carrot fly (*Chamaepsila rosae*), a small black-bodied fly, affects many members of the Apiaceae family, including celery, parsnip, parsley, carrot, and other carrot-family herbs [199]. Plant roots attacked by carrot fly larvae are destroyed, causing fatal damage to affected plants. Bacterial diseases that infect plants through wounds (soft rot or parsnip blight) [200] are the main reason why wounded roots are difficult to store, especially in carrot production [201]. Previous studies revealed that breeding resistant varieties is an effective method of mitigating the effects of carrot fly infestation [202, 203].

The carrot weevil (*Listronotus oregonensis*) is a pest of parsley, carrots, and celery. When carrots are attacked by the carrot weevil, only the ribs of the leaves and stalks are left [140]. This pest causes significant damage to agricultural production and cannot be effectively controlled. At present, no resistant varieties are available [204].

Carrot willow aphid (*Cavariella aegopodii*) is a widespread temperate species that feeds on members of the Apiaceae family [205]. The carrot willow aphid causes direct and indirect damage to plants. Direct damage is mainly caused when the aphid draws juice from plant leaves [206], and indirect damage is caused by the transmission of viral diseases, such as *Carrot red leaf virus* (CRLV), *Parsnip mosaic virus* (PMV), and *Parsnip yellow fleck virus* (PYFV). The main method of controlling carrot willow aphid infestation in agricultural production is the application of pesticides.

Celery fly (*Euleia heraclei*) is a small brown-winged, green-eyed European fly, whose larvae are leaf miners that attack celery and parsnips [207]. These pests burrow inside and destroy the leaves of celery and parsnip, and infested plants show large yellow or brown blotches that are approached by a short gallery [208]. Removing the affected leaves or plants is an effective way of controlling celery fly infestation.

Aphids (Aphidoidea), armyworm (*Mythimna separata*), and cutworms (*Agrotis* spp.) affect many Apiaceae plants, especially fennel plants. Aphids are soft-bodied insects that cause discoloration of leaves, necrotic spots, and stunted growth. The use of resistant varieties and insecticides can effectively control the spread of aphids [209]. The application of *Bacillus thuringiensis* efficiently blocked the spread of armyworm [210]. Cutworms mainly attack the roots of plants, cutting off the transport of water and nutrients between the roots and the aboveground parts. Field management is the main measure used to prevent and control the occurrence of cutworms. These three pests also harm parsley [211].

Beet armyworm (*Spodoptera exigua*) is a pest that is difficult to control and affects celery and celeriac [212]. However, celery cultivars ‘K-26[1]’, ‘K-I08[3]2’, ‘K-I28’, ‘F-128[3]1’, and ‘F-128[4]’ with resistance against beet armyworm have been identified [213]. Moreover, plants with resistance to fusarium yellows displayed a significant increase in beet armyworm resistance [214]. Beet armyworm-resistant cultivars were obtained from 13 cultivars of varieties *rapaceum*, *dulce*, and *secalinum* in 1991 [215].

### Late-bolting breeding

Early bolting significantly decreases the quality and yield of Apiaceae vegetables [216], such as carrot, celery, and parsley [217]. The demand of *Apium* species for a cold period is affected by their environments and genetics [207]. Wohlfeiler *et al*. [218] revealed that the vernalization requirement of carrot was controlled by a multiallelic digene. Previous studies found that annual and biennial celery cultivars bolt easily, and cultivars with strong bolting resistance are rare [169, 219]. A single locus, *Hb*, was identified from *F*_2_ hybrids and found to control the bolting time of celery [220]. Slow-bolting celery cultivars ‘Florida Sloblot M68’ [221] and ‘Juventus’ [222] were generated by single selection and crossing, respectively.

### Molecular marker-assisted breeding

Modern molecular markers include amplified fragment length polymorphisms (AFLPs), simple sequence repeats (SSRs), PCR-based markers, and inter-simple sequence repeat (ISSRs) [223]. These molecular markers have been widely used in breeding members of the Apiaceae family. Que *et al*. [8] summarized the application of molecular markers (polymerase chain reaction (RAPD), AFLPs, quantitative trait locus (QTL) and SSRs) in carrot research, including genetic diversity, population structure, and identification of the difference between CMS and fertile carrots. In celery, RAPD markers were used to explore the genetic diversity of 23 celery cultivars and classify 40 celery varieties from the major regions of China, which showed that celery may be divided into four groups, 12 varieties, and three cultivated types (salad, turnip, and cutting celery) [224, 225]. AFLP technology was used to identify 245 polymorphic sites in 24 celery cultivars using eight AFLP primers [75]. Moreover, five ISSR primers were used to study the genetic diversity of 105 celery accessions, which were classified into five groups [75]. A study of the linkage relationships of 34 markers in celery showed that they were distributed in eight linkage groups, including 21 restriction fragment length polymorphisms (RFLPs), 11 isozymes, and 2 morphological traits, and the total covered length was 318 centimorgans (cM) [226]. In 1995, *F*_2_ population genetic linkage maps of two celery varieties were constructed; these maps contained 29 RFLPs and 100 RAPDs, and they covered a total length of 803 cM [227]. Expressed sequence tags (EST)-SSR fingerprinting, including eight SSR markers, was used to explore the genetic diversity of 11 celery varieties [228]. RNA-seq technology was used to identify 1939 and 2004 SSRs in the ‘Ventura’ and ‘Jinnan Shiqin’ varieties, respectively [229, 230]. In coriander research, many molecular makers, including RAPDs, ISSRs, and SSRs, were also used alone or in combination to explore the genetic diversity of coriander varieties [231–233]. Transcriptome analysis of different tissues of coriander identified 9746 SSRs [234]. In addition, 120 primers were randomly selected to verify 14 coriander accessions in India [234]. Apart from the three plants mentioned above, molecular markers were also widely used to study other Apiaceae species. For example, SSRs and AFLPs were used to investigate the genetic diversity of *Eryngium alpinum* [235, 236]. Single-nucleotide polymorphism (SNP) was used to investigate the genetic diversity and population structure of 78 Western type open-pollinated carrot cultivars [237]. Transcriptome sequencing for high-throughput SNPs revealed that Western carrots may originate from Eastern carrots. The reduction in genetic diversity in Western cultivars due to domestication bottleneck/selection may have been offset by introgression from wild carrot [238]. In addition, ISSR markers were used to determine the phylogenetic relationships among the taxa of *Johrenia* [239–241].

### Transgenic breeding

Agricultural biotechnologies use different techniques to modify the genetic structure of plants to produce genetically modified plants [242]. Transgenic technology can be used to improve plant traits (yield and quality) and solve agricultural problems (biotic and abiotic stresses) [243]. Transgenic systems have been established for only a few Apiaceae vegetables, including carrot and celery. Permyakova *et al*. [244] established transgenic carrot lines overexpressing the *cfp10*, *esat6*, and *dIFN* genes (encoding deltaferon) from *Mycobacterium tuberculosis*, which produce CFP10-ESAT6-dIFN protein in the roots of transgenic carrots, by *Agrobacterium*-mediated transformation. It is most important to emphasize that this genetically modified carrot does not induce immune responses in mice and has no side effects [244]. In addition, transgenic carrot plants expressing human interferon α-2b have been generated [245, 246]. Moreover, in carrot, combined expression of lipid transfer protein (*ltp*) and chitinase (*chi-2*) genes enhanced resistance to foliar fungal pathogens [247–249]. Tan [250] revealed that overexpression of the *AgFNS* gene from purple celery increased apigenin content and decreased anthocyanin content in transgenic celery. Ding *et al*. [251] found that *AgZDS*, a gene encoding ζ-carotene desaturase, increases lutein and β-carotene contents in transgenic *Arabidopsis* and celery. Wang *et al*. [252] reported that AgMYB12, a novel R2R3-MYB transcription factor, regulates apigenin biosynthesis by interacting with the *AgFNS* gene in celery. Overall, the application of genetically modified Apiaceae species will accelerate the breeding of Apiaceae vegetables.

## Genome editing in Apiaceae vegetables

The CRISPR/Cas9 system has been used for targeted mutagenesis in plants, including gene knockout, multiplex gene editing, and insertion and deletion of large fragments [253–255]. A previous study knocked out the carrot gene encoding flavanone 3-hydroxylase (*F3H*), a critical gene for anthocyanin biosynthesis, by genome editing [256]. The results showed that the purple callus in which CRISPR/Cas9 vectors targeted the *F3H* gene became discolored [256, 257]. This gene editing system was also used to knock out other Apiaceae vegetable genes, including carrot *GGred* (geranylgeranyl diphosphate reductase), *LCYE* (lycopene ε-cyclase), *CENH3* (centromeric histone H3), and *DcCCD4* (carotenoid cleavage dioxygenases) [256, 258, 259]. Xu *et al*. [260] also established a stable gene-editing system in carrot, and the system could be used for generating stable gene-edited carrot plants.

## Nanoparticles in Apiaceae plants

Based on previous studies of nanoparticles, it has become evident that nanotechnology can play a vital role in agricultural production, especially regarding gene modification and pest control [261, 262]. Although fertilizers are very important to vegetable crops at all stages, most fertilizers are wasted due to leaching and degradation by various factors. Thus, it is necessary to reduce nutrient waste and increase crop yield through the use of nanomaterials [263]. Nanofertilizers could be more effective than conventional fertilizers because they are capable of releasing nutrients to plants on demand when necessary [264, 265]. At present, the application of nanotechnology is still its infancy in vegetables and medicinal plants of Apiaceae. However, a recent study found that nano-enhanced ammonium bicarbonate increased celery yield and reduced fertilizer requirements [266, 267]. This area of research also provides a new way to perform gene manipulation and expression regulation in plant cells or tissues [268, 269]. In comparison with the widely used *Agrobacterium*-mediated transformation method, nanotechnology can be used to deliver chemicals, proteins, and nucleotides to confer targeted traits on non-genetically modified plants [270].

**Table 2:** Genome information on five sequenced Apiaceae plants.

**Species**	**Source**	**Genus**	**Gene size (Gb)**	**Number of genes**	**Website link**
*Coriandrum sativum*	Bio2RDF	Coriandrum	2.13	40 747	http://cgdb.bio2db.com/databases.html#
*Daucus carota*	NCBI	*Daucus*	0.41	37 099	https://www.ncbi.nlm.nih.gov/genome/?term=Daucus+carota
*Foeniculum vulgare*	NCBI	*Foeniculum*	0.99	43 936	https://www.ncbi.nlm.nih.gov/genome/?term=Foeniculum+vulgare
*Oenanthe javanica*	NCBI	*Oenanthe*	1.28	42 270	https://www.ncbi.nlm.nih.gov/genome/?term=Oenanthe+javanica
*Apium graveolens*	NCBI	*Apium*	3.25	31 326	https://www.ncbi.nlm.nih.gov/genome/11000

## Omics research in vegetables and medicinal plants of Apiaceae

### Genomics

Omics research attempts to comprehensively understand the biological molecules in an organism at a particular functional level, such as the genome, transcriptome, or proteome [271–273]. Apiaceae is a large angiosperm family that includes many medicinal, edible, and spice species, which play important roles in daily life around the world [272]. A gene-editing system for carrots was established and used to determine the inheritance of anthocyanin sites in carrots, providing new ideas and methods for transgenic carrot breeding [260, 274].

Although the members of Apiaceae have a wide geographical distribution and rich nutritional and medicinal value, little research has been performed on the genomes of Apiaceae species [275]. Here, we summarize genomic information for five representative species that have been sequenced, assembled, and annotated well, including coriander (2*n* = 2*x* = 22), celery (2*n* = 2*x* = 22), and carrot (2*n* = 2*x* = 18). In 2014, Xiong’s group established CarrotDB, a genomic and transcriptomic database for carrot [276, 277]. In 2016, Simon’s group published a high-quality carrot genome sequence assembly (421.5 Mb) with the N50 scaffold length of 64.5 kb [278]. In 2018, Feng *et al*. [279] established CeleryDB, a celery genome database. Later, Li *et al*. [280] published the genome sequence of celery and identified important functional genes. More recently, a high-quality celery genome sequence, with N50 scaffold length of 289.78 Mb, was made available [281]. The coriander genome sequence was published in 2020. The total assembled coriander genome size is 2.13 Gb, which is divided over 6186 scaffolds with an N50 scaffold length of 160.99 Mb [282].

In addition to celery, carrot, and coriander, the genomes of two other Apiaceae plants have been sequenced. *Oenanthe javanica* (Blume) DC., a Chinese herbal medicine, belongs to the Apiaceae family [283]. The *O. javanica* genome was published in 2021 [284]. The assembled *O. javanica* genome contains 149 923 scaffolds, the size of the assembled genome is 1.28 Gb, and the N50 scaffold length is 13.093 Mb. Fennel, belonging to the genus *Foeniculum* in Apiaceae, is a Chinese herbal plant used to treat various diseases [285]. The assembled genome of fennel consists of 300 377 scaffolds, the total length of the genome is 1010.97 Mb, and the N50 scaffold length is 18.88 Mb. Many studies have revealed that plant genomes contain abundant repeat sequences. Genomic sequences and annotation have provided important information that has contributed to studies of the functions of genes involved in regulating the yield and quality traits of horticultural crops [286]. The further study of important gene functions and breeding, as well as comparative genomic analysis of Apiaceae, will provide new methods for genetic and breeding research using Apiaceae vegetable crops and medicinal plants. Genome information on five Apiaceae plants is shown in Table 2.

### Transcriptomics

Transcriptome data are widely used in gene expression analysis, gene function discovery, and molecular marker development [287, 288]. Although the Apiaceae family has a large number of members, some Apiaceae vegetable crops have undergone transcriptome analysis [8, 277–284, 289–290]. Besides, transcriptome technology has also been applied in research on stress response [291], root development [292], and lignin biosynthesis in carrot [293].

Moreover, transcriptome analysis has been used widely in celery research. Jia *et al*. [294] revealed the mechanism of formation of lignin and hormones based on transcriptome profiles of celery at different developmental stages. Through transcriptome analysis, Liu *et al*. [295] found that multiple genes controlling hormone synthesis in celery were associated with leaf development. Li *et al*. [296] demonstrated the relationship between related gene expression profiling and accumulation of β-carotene in celery leaves and petioles using transcriptome analysis. Jiang *et al*. [297] identified the response genes of *Oenanthe javanica* under abiotic stress through transcriptome assembly and gene annotation. Tan and colleagues [298] analyzed temperature stress response genes by *de novo* assembly and transcriptome characterization in *Cryptotaenia japonica*. Li *et al*. [299] also identified abiotic stress-related AP2/ERF transcription factors by transcriptome sequencing and analysis of parsley.

Transcriptomics have also been applied to study Apiaceae plants used in Chinese herbal medicine. For example, transcriptome analysis of different tissues from *Ferula assa-foetida* revealed candidate genes for terpene and phenylpropyl metabolism [300]. In conclusion, the application of transcriptomics allows researchers to explore the phenotypic characteristics of vegetables and medicinal plants in Apiaceae and the physiological functions of Apiaceae genes.

### MicroRNAs

MicroRNAs (miRNAs) are endogenous small RNAs that play important roles in regulating plant growth and development [301, 302]. In the process of plant development, miRNAs play key roles at every major stage [302–304]. Drikvand *et al*. [305] identified three miRNAs (csa-miR162, csa-miR169, and csa-miR399) in coriander and found that the target genes of these miRNAs displayed differential expression in seed and leaf samples. A total of 431 and 346 miRNAs were identified in celery varieties ‘Ventura’ and ‘Jinnan Shiqin’, respectively, and 6 of these miRNAs were found to be involved in responses to cold and heat stresses [229]. Najafabadi *et al*. [306] identified the top five miRNAs (2919, 5251, 838, 5021, and 5658) involved in the biosynthesis and regulation of terpenes in *Ferula gummosa*. Jia *et al*. [307] identified 344 conserved miRNAs associated with leaf development in celery. Jiang and colleagues also identified microRNAs affected by abiotic stress in celery [308]. Bhan *et al*. (2019) surveyed the miRNAs in two carrot variants with different colors (orange-red and purple) using RNA-seq, leading to the validation of 2 novel miRNAs and 11 known miRNAs [309]. Recently, the responses to water stress were investigated using integrative genome, transcriptome, miRNA and degradome analysis in *O. javanica* [284].

### Proteomics

Proteomics is now considered one of the most important ‘post-genomic’ approaches to help us understand the function of genes. In fact, some genomics companies have launched large-scale proteomics projects [310]. Proteomics, of course, is widely used to study the Apiaceae plants. Huang *et al*. [311] performed proteomic analysis of temperature stress-responsive proteins in celery leaves and identified 71 temperature-responsive proteins. Khodadadi *et al*. [312] elucidated the response mechanism in drought-sensitive and -tolerant genotypes of fennel leaf using a gel-free/label-free proteomic technique, and further analysis revealed that drought stress may limit photorespiration by reducing the activity of cobalamin-independent methionine synthase in drought-sensitive genotypes. Bai *et al*. [313] reported the precise mechanism by which asafoetida extract influenced the growth of *Pleurotus ferulae* mycelium using comparative proteomic analysis, and the results showed that asafoetida extracts significantly affected the growth and metabolism of *P. ferulae* [313]. Comparative proteomic analysis also provides new insights into gene mining in carrot plants [314, 315].

### Metabolomics

Metabolomics encompasses all chemical reactions occurring in cells. GC–MS technology has been used for metabolite profiling since the early 1990s [316]. Plant metabolites have been used as chemical markers to distinguish differences among vegetables and medicinal plants of the Apiaceae family [317]. In carrot research, metabolomics analysis revealed that wild and cultivated carrots showed differences in metabolites [318] that were consistent with their genotypes. Identification of the *WtDcTPS1* gene, which is involved in the synthesis of geraniol in wild carrot, was achieved by metabolomics analysis [319].

NMR-based metabolomics has been used to discriminate celery from different geographical origins [320, 321]. Based on UHPLC–QTOF–MS/MS metabolomics analysis, nine chemical markers were used to distinguish *Radix Angelica sinensis* samples from different regions [322, 323]. *Radix bupleuri* is one of the most popular traditional Chinese herbal drugs [324–326]. Studies have shown that *R. bupleuri* protects the liver by interacting with various metabolic processes [327–329]. DG (Danggui, *A. sinensis*) products were found to significantly relieve blood stasis syndrome in rats, and Jiu Danggui was the most effective type [330]. In addition, plant metabolites are involved in the color, taste, and scent of fruits and flowers, and they also contribute to the regulation of various resistance and stress responses [331].

In recent years, environmental scientists have developed practical applications for metabolomics. In carrot research, Koutouan *et al*. [181] reported a link between leaf secondary metabolites and resistance to *Alternaria dauci*. Another recent study identified genes and metabolites in important biological pathways that may regulate selenium tolerance in celery [332]. Many plants of the Apiaceae family are used as condiments or vegetables, and some of them have medicinal properties that may be related to secondary metabolites [333]. In summary, metabolomics analysis is an important method for the in-depth study of the physiological and biochemical processes of vegetables and medicinal plants in Apiaceae, and could provide new possibilities for human use.

### Functional genes involved in the synthesis of nutraceuticals in Apiaceae vegetables and medicinal plants

Vegetables and medicinal plants in the Apiaceae family are good sources of many secondary metabolites, such as carotenoids, anthocyanins, terpenes, and dietary fiber [118, 334]. Information on identified functional genes in some Apiaceae plants is shown in Table 3.

**Table 3:** Information on identified functional genes in some Apiaceae plants.

**Species**	**Gene name**	**Gene expression status**	**GenBank**	**Function**
*Daucus carota*	*PSY*	Overexpression	NM_001329177.1	Increased content of carotenoids [340]
	*PDS*	Overexpression	NM_001329175.1	Produced β-carotene and α-carotene [341]
	*ZISO*	Overexpression	XM_017363269.1	Produced β-carotene and α-carotene [341]
	*ZDS*	Overexpression	NM_001329165.1	Produced β-carotene and α-carotene [341]
	*CRTISO*	Overexpression	XM_017392673.1	Produced β-carotene and α-carotene [341]
	*LCYB*	Overexpression	NM_001329160.1	Produced β-carotene and α-carotene [342]
	*LCYE*	Overexpression	NM_001329163.1	Produced β-carotene and α-carotene [342]
	*CYP97A3*	Overexpression	JQ655297.1	Decreased content of α-carotene in roots [345]
	*F3H*	Overexpression	XM_017385173.1	Regulated biosynthesis of anthocyanins [256]
	*UCGalT1*	Overexpression	KP319022.1	Regulated biosynthesis of anthocyanins [371]
	*MYB6*	Overexpression	XM_017379690.1	Regulated biosynthesis of anthocyanins [368]
	*MYB7*	Overexpression	XM_017385289.1	Regulated biosynthesis of anthocyanins [274]
	*MYB113*	transcriptome	XM_017383803.1	Regulated biosynthesis of anthocyanins [271]
	*USAGT1*	Overexpression	KT595241.1	Regulated biosynthesis of anthocyanins [370]
	*bHLH*	Overexpression	QEA09235.1	Colored with carrot taproot anthocyanin [372]
	*GST*	Overexpression	XM_017389912.1	Colored with carrot taproot anthocyanin [372]
	*TPS04*	Overexpression	XM_017390437.1	Produced α-terpineol, sabinene, β-limonene, β-pinene, myrcene [388]
	*TPS26*	Recombinant protein expression in *Escherichia coli*	XM_017390438.1	Regulated monoterpene production [289–291]
	*TPS27*	Recombinant protein expression in *Escherichia coli*	KZM99345.1	Regulated monoterpene production [289–291]
	*TPS54*	Recombinant protein expression in *Escherichia coli*	KZM99341.1	Formed sabinene [388]
	*TPS55*	Recombinant protein expression in *Escherichia coli*	KZM99344.1	Regulated monoterpene production [387–389]
	*TPS1*	Recombinant protein expression in *E. coli*	DcTPS58617	Synthesized (*E*)-β-caryophyllene α-humulene [390]
	*TPS2*	Recombinant protein expression in *Escherichia coli*	XM_017389213.1	Synthesized monoterpene synthase with geraniol [390]
	*atp6*	Overexpression	JQ248574.1	Associated with carrot male sterility [160]
	*atp9*	Overexpression	AJ009982.1	Associated with carrot male sterility [165]
	*DFR2*	Overexpression	AF184272_1	Involved in anthocyanin synthesis [8]
	*UFGT*	Overexpression	XM_017392428.1	Involved in anthocyanin synthesis [8]
	*FLS1*	Overexpression	XM_017372509.1	Involved in anthocyanin synthesis [8]
	*LDOX2*	Overexpression	AF184274.1	Involved in anthocyanin synthesis [8]
	*MYB2*	Overexpression		Participated in anthocyanin synthesis regulation in purple celery [373]
*Apium graveolens*	*γTRPS*	Recombinant protein expression in *Escherichia coli*	KF700699.1	Catalyzed the conversion of geranyl diphosphate [392]
	*FNS*	Overexpression	AY817676.1	Increased content of apigenin, decreased content of anthocyanin in petiole of transgenic celery [252]
*Coriandrum sativum*	*LINS*	Recombinant protein expression in *Escherichia coli*	KF700700.1	Catalyzed conversion of geranyl diphosphate [392]

#### Carotenoids

Carotenoids are natural pigments that are widely distributed in photosynthetic organisms and may provide health benefits [335, 336]. The first committed step in carotenoid biosynthesis is catalyzed by phytoene synthase (PSY) [337–339]. Overexpression of *PSY* increased the content of carotenoids in transgenic plants [340]. The PSY product, 15-*cis*-phytoene, is further desaturated and isomerized to form all-*trans*-lycopene by phytoene desaturase (PDS), 15-cis-ζ-carotene isomerase (Z-ISO), ζ-carotene desaturase (ZDS), and cis-trans-isomerization (CrtISO) [341]. Next, lycopene cyclases, including lycopene cyclase β (LCYB) and lycopene cyclase ε (LCYE), are involved in producing β-carotene and α-carotene [342]. β-Carotene, known as the orange-red pigment in carrots, has been shown to account for 80% of the total carotene content in this vegetable [343]. Moreno *et al*. [344] revealed that *DcLcyb1* plays an essential role in the accumulation of β-carotene in carrot plants. Arango *et al*. [345] found that overexpression of *CYP97A3* in orange carrots significantly decreased the content of α-carotene in roots without significantly changing the content of α-carotene in leaves. Analysis of domesticated varieties and wild carrot accessions revealed a significant genomic region that contains the *Or* (Orange) gene, which is a candidate for carotenoid presence in carrot [346]. Coe *et al*. [347] have revealed that *Or* and *CH* are likely involved in controlling the accumulation of β-carotene and modulated carotenoid flux in carrot. Wang *et al*. [348] found that the expression profiles of the genes related to carotenoid biosynthesis were closely related to carotenoid content in carrots with different colors. Zhang *et al*. [349] found that the carotenoid contents and expression of related genes were affected by drought stress in carrot taproots. Then, Li *et al*. [350] reported that *Arabidopsis* plants hosting the *DcBCH1* gene, encoding non-heme carotene hydroxylase (BCH), improves tolerance to drought in transgenic plants. BCH is a key regulatory enzyme in the β-branch of the carotenoid biosynthesis pathway. In addition, multiple paralogs of carotenoid pathway genes have been identified in carrot, suggesting that different paralogs are involved in the precise temporal regulation of carotene synthesis in different tissues, developmental stages, and environmental conditions [351].

The expression of carotenoid pathway genes also increases the accumulation of other pigments in Apiaceae vegetables [352–356]. In celery, the relative expression levels of *AgPSY1* and *AgLCYE* in the ‘Ventura’ cultivar were significantly higher than those in the ‘Liuhe Yellow Heart Celery’ cultivar [357]*_._* Furthermore, transcriptome profiling of biosynthesis genes and β-carotene content in the leaf blades and petioles of celery demonstrated that *AgPSY1*, *AgCRTISO2*, and *AgBCH1* may play important roles in the accumulation of β-carotene [296]. Similar results have been reported in carrot [290, 358, 359]. Ding *et al*. [360] found that the expression levels of *AgLCYB* and *AgPSY2* genes were significantly correlated with lutein and β-carotene contents in yellow celery. Yin *et al*. [361] and Ding *et al*. [360] demonstrated that overexpression of the genes *AgLCY-ε* and *AgZDS*, encoding lycopene epsilon cyclase and ζ-carotene desaturase, increased lutein and β-carotene accumulation in transgenic *Arabidopsis*.

#### Anthocyanins

Anthocyanins are phenolic compounds that are synthesized via the phenylpropanoid pathway and add pigmentation to several organs and tissues of many plant species [362]. Anthocyanins protect plants from UV radiation, contribute to plant adaptation to different abiotic and biotic stresses, and delay plant senescence [363, 364]. In addition, anthocyanins promote various health benefits due to their antioxidant effects and anti-inflammatory properties [365]. Research on anthocyanins in Apiaceae has mainly focused on a few species, including carrot and purple celery.

In carrot research, previous studies have provided many sources of information on anthocyanins in carrot, such as the content of anthocyanins of different varieties (purple, yellow and orange carrots), structural genes encoding key enzymes, and transcription factors regulating anthocyanin biosynthesis [274, 366–371]. Furthermore, Chialva *et al*. [253] identified long non-coding RNAs (lncRNAs) involved in regulating anthocyanin biosynthesis in taproots. A transcriptome analysis strongly suggested that transcription factors bHLH and GST are involved in anthocyanin pigmentation in carrot roots [372].

Recent studies found that transcription factors AgMYB1/AgMYB2 and OjMYB1 are involved in the regulation of anthocyanin biosynthesis in purple celery (*Apium graveolens*) and *Oenanthe javanica*, respectively [373–376]. AgMYB12, a R2R3-MYB transcription factor, regulates apigenin biosynthesis in transgenic celery. Overexpression of AgMYB12 in celery improved the accumulation of apigenin by interacting with the *AgFNS* [252, 377]. Feng *et al*. [378] demonstrated that the gene *AgUCGalT1*, encoding galactosyltransferase, regulated anthocyanin galactosylation in purple celery.

**Figure 1:**
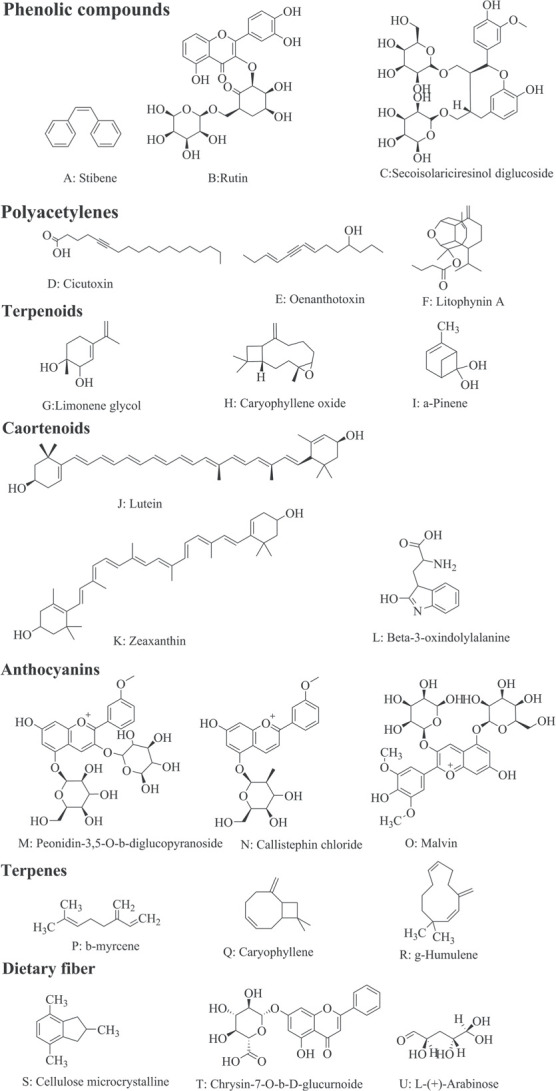
Molecular structure of main bioactive compounds of Apiaceae plants.

#### Terpenes

Terpenes are an important group of secondary metabolites that affect taste and flavor [379]. Terpene synthases (TPSs) are catalysts responsible for the formation of sesquiterpenes, monoterpenes, and diterpenes [380–382], which are widely distributed in many plants [382–385]. In carrot research, Keilwagen *et al*. [386] identified 65 putative *TPS* family genes. A previous study identified a carrot *TPS* gene cluster on chromosome 4 that was found to be related to monoterpene production, including *DcTPS04*, *DcTPS26*, *DcTPS27*, *DcTPS54*, and *DcTPS55* [387–389]. *In vitro* enzyme assays of DcTPS54 and DcTPS04 showed that DcTPS54 is responsible for the formation of sabinene, whereas DcTPS04 is involved in producing the major products α-terpineol, sabinene, β-limonene, β-pinene, and myrcene [388]. Analysis by Yahyaa *et al*. [390] revealed the function of two TPSs, the sesquiterpene synthase DcTPS1 and the monoterpene synthase DcTPS2. DcTPS1 is responsible for the synthesis of (*E*)-β-caryophyllene and α-humulene in carrot [391]. In *Coriandrum sativum*, two TPSs, the recombinant proteins CsγTRPS and CsLINS, were found to catalyze the conversion of geranyl diphosphate [392]. Song *et al*. [282] first systematically identified TPS family genes in *C. sativum.*

#### Dietary fiber

Dietary fiber in plants is classified as soluble or insoluble. Soluble fiber is found in many plants, including carrots, broccoli, onions, barley, bananas, berries, apples, and pears. Insoluble fiber is found in whole grain, wheat, bran, nuts, seeds, and some fruits and vegetables [393, 394]. Dietary fiber plays an important role in moderating the postprandial insulin response and reducing cholesterol and the incidence of heart disease, among other beneficial effects [395]. The plant cell wall, including the primary and secondary wall, which contain lignin and cellulose, is the source of most of the dietary fiber in plants [396].

Hormones play important roles in lignin biosynthesis in celery [397–399] and carrot [400–403]. Transcription factors were important regulators of lignin biosynthesis in celery and carrot [404, 405]. Hypoxia, caused by elevated CO_2_ concentration also affected lignin content in celery and carrot [315, 406, 407]. The chemical molecular structures of the main bioactive compounds in Apiaceae plants are shown in Fig. 1.

## Conclusions and future perspectives

Vegetables and medicinal plants are essential foods in human diets and health care, and can provide various necessary nutrients and nutraceuticals. With the strengthening of people’s health consciousness, the diversification, quality, nutritional value, and medicinal value of vegetables and medicinal plants are increasing. Vegetable and medicinal plant research has become increasingly important. In this review, we summarize the origin of Apiaceae plants, common vegetables, and medicinal plants of Apiaceae, bioactive compounds, medicinal applications, traditional and molecular breeding, functional genes, omics analysis, and other aspects. Although a number of Apiaceae plants have been discovered, few members have been utilized for the specific compounds they contain. In the future we should collect Apiaceae germplasm. Omic analysis (transcriptome, genome, and metabolome) is used to explore gene information and bioactive substances in Apiaceae plants. Integration of DNA molecular markers and genome-wide association analysis (GWAS) explores the relationship between genotypes and phenotypes and mine the variation in genomic loci associated with the important agronomic traits. Molecular breeding, including genetic transformation and the CRISPR-Cas9 gene editing system, will be widely used in Apiaceae plant breeding.

### Improving breeding level

In crop breeding, excellent varieties have been selected and planted for quality, size, and biotic and abiotic tolerances. Although a few members of the Apiaceae, such as carrot, coriander, and celery, are the most widely grown vegetable crops in the world, their cultivars are insufficient to meet the demand from health-conscious consumers looking for more vegetables and medicinal plants among the Apiaceae. China is rich in wild germplasm resources of Apiaceae. In the future, the purpose of research is to collect and domesticate wild germplasm resources, and increase the exploration and utilization of wild germplasm resources to create more cultivated varieties. In addition, more effective breeding platforms and technology fully combine traditional breeding programs with modern molecular technologies should be established. Molecular markers, GWAS, genetic modification (usually using CRISPR-Cas9 technology to create non-transgenic mutant plants), and nanotechnology should be widely used to guide traditional breeding or molecular breeding.

### Mining functional genes

Plant genomes and transcriptomes have been used to explore gene information. Combined transcriptome and metabolome analysis has explored bioactive compounds, functional genes, and transcription factors. The yeast one-hybrid and yeast two-hybrid systems are widely recognized as valuable and straightforward techniques to study interactions between transcription factors and between DNA and transcription factors. Integration of DNA molecular markers and GWAS explore the relationship between genotypes and phenotypes, and mine the variation in genomic loci associated with the important agronomic traits and detected key genes.

### Extraction and utilization of bioactive ingredients

Vegetables and medicinal plants in Apiaceae are an excellent source of secondary metabolites, which specifically modulate health-maintaining processes. However, the sample extraction techniques severely block the isolation and extraction of individual secondary metabolites in Apiaceae plants, which severely restricts the development of traditional Chinese medicine. In addition, the pharmacological mechanisms of active ingredients in many vegetables and medicinal plants of Apiaceae are still unclear due to the lack of the animal studies and clinical trials. With the innovations of new technology and the development of molecular biology, research on bioactive ingredients mainly focuses on their isolation and extraction, structure analysis, metabolic pathway analysis, and molecular mechanisms.

### Omics of vegetables and medicinal plants in Apiaceae

The family Apiaceae is in the major group flowering plants, and contains >3700 species in 434 genera. However, there are only a few species with available genomes. Innovations in sequencing technology and reduction of sequencing costs provide a great opportunity for studying Apiaceae plant genomes. High-quality genomes of Apiaceae plants contribute to faster and more accurate understanding of genome structures, functional gene information, and other sequences. Moreover, comparative genomics research is commonly used to explore the origin and evolution history of vegetables and medicinal plants in Apiaceae. The applications of GWAS help us identify SNPs and InDels among the different varieties of Apiaceae crops. In addition, comprehensive transcriptome, proteome, and metabolome analysis promotes discoveries in expression patterns and gene function and structure, as well as metabolite components in vegetables and medicinal plants in Apiaceae.
